# Erythrocyte intracellular Mg^2+^ concentration as an index of recognition and memory

**DOI:** 10.1038/srep26975

**Published:** 2016-06-02

**Authors:** Wenxiang Xiong, Yaru Liang, Xue Li, Guosong Liu, Zhao Wang

**Affiliations:** 1 MOE Key Laboratory of Protein Sciences, Department of Pharmacology, School of Medicine, Tsinghua University, Beijing 100084, P.R. China

## Abstract

Magnesium (Mg^2+^) plays an important role in the neural system, and yet scarcely any research has quantitatively analyzed the link between endogenous Mg^2+^ level and memory. Using our original technique, we measured erythrocyte intracellular ionized Mg^2+^ concentration (RBC [Mg^2+^]_i_), which linearly correlated to recognition and spatial memory in normal aging rats. In the brain, RBC [Mg^2+^]_i_ significantly correlated to hippocampus extracellular fluid Mg^2+^ concentration, and further correlated to hippocampal synapse density. Elevation of Mg^2+^ intake in aged rats demonstrated an association between RBC [Mg^2+^]_i_ increase and memory recovery. The therapeutic effect of Mg^2+^ administration was inversely correlated to individual basal RBC [Mg^2+^]_i_. In summary, we provide a method to measure RBC [Mg^2+^]_i_, an ideal indicator of body Mg^2+^ level. RBC [Mg^2+^]_i_ represents rodent memory performance in our study, and might further serve as a potential biomarker for clinical differential diagnosis and precise treatment of Mg^2+^-deficiency-associated memory decline during aging.

Magnesium (Mg^2+^) is the fourth most abundant ion in the body and the second most abundant ion in the cells. Mg^2+^ is a cofactor for more than 300 enzymatic reactions, responsible for conducting biomolecule synthesis/hydrolysis, signal transmission, and gene expression. It also binds to ATP to maintain its stability, and is therefore essential for energy metabolism. Mg^2+^ participates in important vital activities, such as cell proliferation, neuron spiking, and muscle contraction/relaxation, among others^1^,^2^,^3^,^4^,^5^.

In the neural system, Mg^2+^ also plays important roles. It competes with calcium (Ca^2+^) to mediate N-methyl-D-aspartic acid receptor (NMDAR)-dependent neural activity. Although Mg^2+^ blocks NMDAR at resting membrane potential, our previous study showed that, chronic elevation of Mg^2+^ level in hippocampal neuron culture medium upregulated NMDAR signal transmission and enhances synaptic plasticity^6^. In our follow-up study, oral administration of magnesium-L-threonate (MgT) to aged rats recovered their behavioral impairments^7^. These findings suggest a crucial role for Mg^2+^ in brain function. However, our previous *in vivo* studies did not show any difference of endogenous Mg^2+^ level between young and aged rats. That means, although exogenous Mg^2+^ administration showed notable therapeutic effects on the memory recovery of aged rats, it was still not clear whether the memory impairment during aging was caused by endogenous Mg^2+^ level changes or not. In fact, all the figures which involved in the therapeutic effect of Mg^2+^ supply enhancement, whatever on the behavioral, cellular or molecular level, lack support of endogenous Mg^2+^ data^7^. It only reported a slight increase in the Mg^2+^ concentration of the cerebrospinal fluid (CSF [Mg^2+^]) after Mg^2+^ administration, in an independent experiment of exogenous Mg^2+^ delivery optimization. Yet, the evidence was far from enough. It requests further studies on the relationship between endogenous Mg^2+^ status and memory performance.

To find an ideal Mg^2+^ marker for brain function and memory is a challenging task. Many Mg pools in the body could serve as potential candidates. The total Mg content in an adult human is about 1 mol: 60~65% of it is contained in the skeleton, 27% in skeletal muscle, 6~7% in other tissues, and 1% in extracellular fluid^1^. In cells, most of the Mg is bound to biomolecules, leaving about 10% as the free ionized form. Previous research has assessed Mg content (ionized and/or non-ionized) in different tissues using various techniques (Supplementary Tables S1 and S2). Unfortunately, many of the potential candidates showed no response to physiological and pathological processing, and therefore could not function as indicators. A few of others had a very limited applicable range. By contrast, RBC [Mg^2+^]_i_ was found to be a potential Mg^2+^ index, which effectively represents body Mg^2+^ loss in the cases of hypertension and/or diabetes^8^,^9^,^10^,^11^,^12^, which led us to focus on the RBC [Mg^2+^]_i_ to study its capability as a body Mg^2+^ marker.

Aging was also associated with RBC [Mg^2+^]_i_ decline^8^. The age related body Mg^2+^ deficiency may be caused by unhealthy dietary pattern^13^,^14^, abnormal Mg^2+^ absorbance/clearance function^15^,^16^,^17^,^18^, insulin resistance and metabolic syndrome^10^,^19^,^20^, and abnormal hormone secretion^21^,^22^. On the other hand, aging was a predictor for memory loss^23^,^24^,^25^,^26^,^27^, while compensation of Mg^2+^ intake could rescue cognitive function in aged animals^7^. These evidences led us to hypothesize that the age related RBC [Mg^2+^]_i_ decline might correspond with memory impairment during aging. Therefore, we used aging rats as a model to research whether RBC [Mg^2+^]_i_ could be a potential memory indicator.

In the present study, we found and confirmed a significant correlation between RBC [Mg^2+^]_i_ and memory performance in aged rats. The level of RBC [Mg^2+^]_i_ directly influenced memory status, when the supply of exogenous Mg^2+^ was changed in aged rats. The effect of abundant Mg^2+^ supply to improve memory was inversely correlated to the individual basal RBC [Mg^2+^]_i_ in aged rats.

## Results

### RBC [Mg^2+^]_i_ indicates body Mg^2+^ loss

For the purpose of studying the correlation between Mg^2+^ and memory, we first evaluated the possibility that RBC [Mg^2+^]_i_ could act as an indicator for body Mg^2+^ status. Mg^2+^ intake deficiency was an ideal model for creating a dramatic Mg^2+^ change in a short term. The Mg^2+^ content in the blood, tissue, and skeleton has shown a decline in this model^28^,^29^,^30^,^31^,^32^,^33^. We postulated that if RBC [Mg^2+^]_i_ was also a body Mg^2+^ marker, it would respond to food Mg^2+^ restriction too.

**Figure 1:**
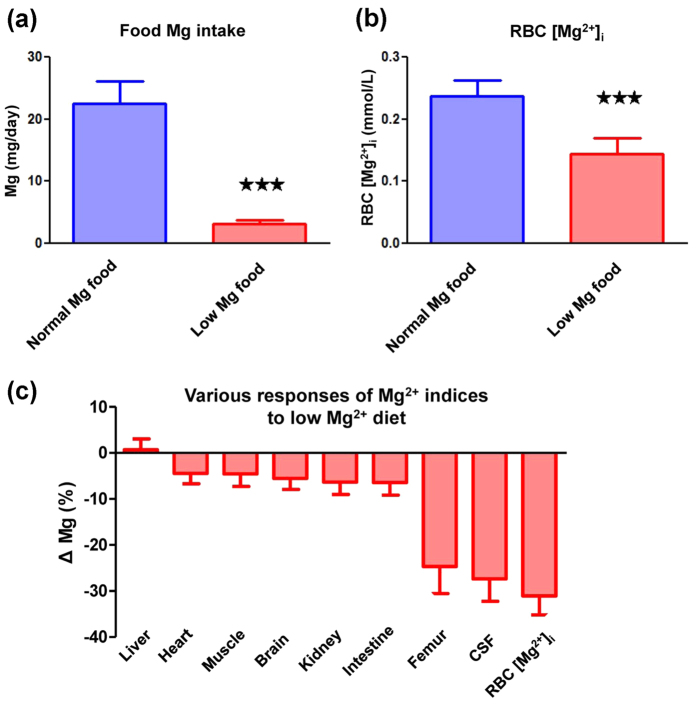
RBC [Mg^2+^]_i_ indicates body Mg^2+^ decline. (**a**) Daily Mg^2+^ intake in normal and low Mg^2+^ diet groups. (**b**) RBC [Mg^2+^]_i_ in normal and low Mg^2+^ diet groups. (**c**) Percentage changes of Mg (Mg^2+^ or total Mg) in tissues, cerebrospinal fluid (CSF), and erythrocytes (RBCs) (calculation was described in Equation (1)). Young rats (3 months), *n* = 10 for each group. Unpaired *t* test, ***p < 0.001.

Feeding the rats with Mg^2+^ deficient food caused a dramatic drop in total Mg intake, from 22.5 ± 3.62 to 3.16 ± 0.56 mg/day (*p* < 0.001; Fig. 1a). A negative value of body Mg retention was found (see Supplementary Fig. S1), indicating a body Mg^2+^ loss. For our interest, the RBC [Mg^2+^]_i_ significantly decreased in the Mg^2+^ restriction group, comparing to the normal Mg^2+^ group (0.14 ± 0.025 vs. 0.24 ± 0.026 mmol/L, *p* < 0.001; Fig. 1b). In other endogenous Mg^2+^ compartments, the total Mg in soft tissues showed changes of less than 7% (Equation (1)) after Mg^2+^ restriction. The skeletal total Mg, which represented the largest exchangeable Mg^2+^ pool in the body, showed a 24.68% decline. The CSF [Mg^2+^] showed a 27.36% decline, which was similar to the RBC [Mg^2+^]_i_ (a 31.05% decline, Fig. 1c). These results in tissue Mg^2+^ pool agreed with previous reports^28^,^29^,^30^,^31^,^32^,^33^.

Therefore, we concluded that RBC [Mg^2+^]_i_ could serve as a body Mg^2+^ marker in rats, which sufficiently indicated the body Mg^2+^ decline. The similar changes in scale seen for CSF [Mg^2+^] and RBC [Mg^2+^]_i_ supported a potential ability of RBC [Mg^2+^]_i_ to represent brain Mg^2+^ levels.

### Aging-associated RBC [Mg^2+^]_i_ loss correlates to memory decline

Our next task was to assess whether RBC [Mg^2+^]_i_, our selected endogenous body Mg^2+^ marker, would show any direct relationship with memory performance. Aging is known as a physiological process that induces both body Mg^2+^ deficiency and memory decline. We studied whether RBC [Mg^2+^]_i_ level and memory status correspond to each other during aging, by assessing behavioral performance and RBC [Mg^2+^]_i_ level in young and aged rats.

**Figure 2:**
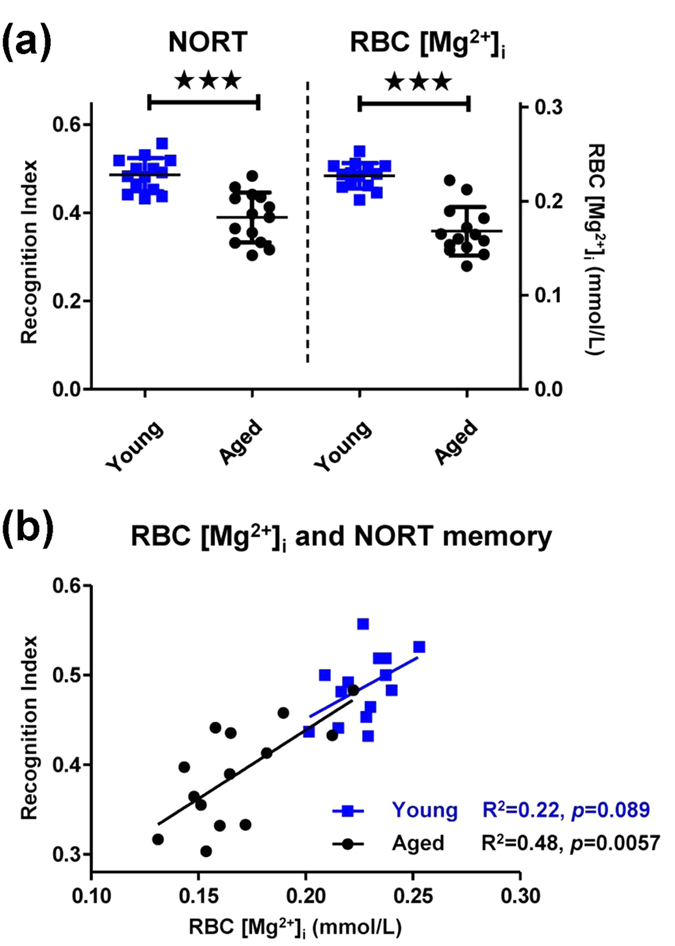
Correlation analysis between RBC [Mg^2+^]_i_ and recognition memory in young and aged rats. (**a**) The novel object recognition test (NORT) recognition index (left) and RBC [Mg^2+^]_i_ (right) of young (3 months) and aged (22 months) rats. (**b**) Linear correlation between RBC [Mg^2+^]_i_ and recognition index in young and aged rats. *n* = 14 for each group. Unpaired *t* test, ***p < 0.001.

In the novel object recognition test (NORT), aged animals showed a significant decrease in recognition score comparing to young group, (*p* < 0.001; Fig. 2a). The aged rats had a greater variation: not all aged animals had memory decline; some individuals retained equal memory to that seen in the young rats. The RBC [Mg^2+^]_i_ levels were significantly lower in aged rats (*p* < 0.001; Fig. 2a), indicating a body Mg^2+^ decline during aging. The variation in individual RBC [Mg^2+^]_i_ levels showed a similar pattern to that seen for the recognition index.

In addition to conducting an intergroup comparison, we also performed a correlation study between individual RBC [Mg^2+^]_i_ and memory in a single group. The RBC [Mg^2+^]_i_ not only corresponded with the recognition index between young and aged groups, but also showed a significant linear correlation with the recognition index in aged individuals (Fig. 2b). It implied that, the value of endogenous Mg^2+^ could represent individual memory: a lower RBC [Mg^2+^]_i_, would indicate a more forgetful aged individual. The correlation between RBC [Mg^2+^]_i_ and recognition index was not significant in the young rats. One explanation for this result might be the lesser dispersion of both the RBC [Mg^2+^]_i_ and recognition index in the young group, which made the data ineffective for a intra-group correlation study.

**Figure 3:**
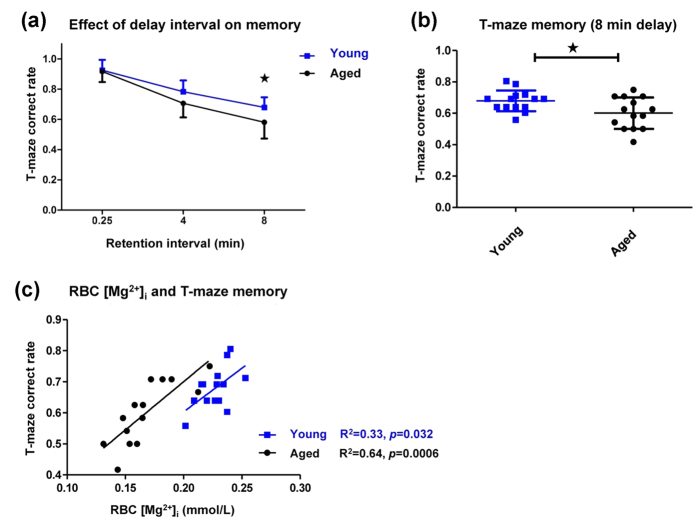
Correlation analysis between RBC [Mg^2+^]_i_ and spatial memory in young and aged rats. (**a**) The T-maze correct choice rate of young and aged rats with different retention intervals. Two-way ANOVA followed by Bonferroni’s *post hoc* test, *p < 0.05. (**b**) The T-maze correct choice rate of young and aged rats with 8 minutes’ retention. Unpaired *t* test, *p < 0.05. (**c**) Linear correlation between RBC [Mg^2+^]_i_ and T-maze correct choice rate (8 minutes retention) in young and aged rats. (The same animals as in Fig. 2 were used).

The correlation between RBC [Mg^2+^]_i_ and spatial working memory was examined by T-maze test in young and aged rats, in order to confirm the correlation studies above. The difficulty of the T-maze task depended on the retention interval between the sample run and the choice run. An extension in the interval was associated with a decrease in the correct choice rate in both rat groups. A significant difference was noticed between the young and aged rats at an 8 minute interval (Fig. 3a). Thus, we chose 8 minutes as the optimized delay interval to conduct subsequent experiments. The same animals used in the NORT experiment were used for the T-maze test here. The RBC [Mg^2+^]_i_ levels of these rats have already been shown in Fig. 2, so we did not illustrate them again.

The young rats performed better than aged rats in the 8 minute T-maze test (Fig. 3b). A linear correlation was observed between RBC [Mg^2+^]_i_ and the correct choice rate in the young rats (Fig. 3c), and the correlation was much stronger in the aged rats (R^2^ = 0.64, *p* = 0.0006).

Again, RBC [Mg^2+^]_i_ and memory appeared to have a tight connection. RBC [Mg^2+^]_i_ was positively correlated with recognition memory as well as spatial working memory in aged animals, indicating that the RBC [Mg^2+^]_i_ might be used to predict memory performance during aging. Aging caused widespread distributions in both RBC [Mg^2+^]_i_ and behavioral performance (Figs 2a and 3b). It revealed that only “being old” did not strictly indicate memory decline, while the decrease in RBC [Mg^2+^]_i_ rigidly matched the memory deficiency.

### Exogenous compensation of RBC [Mg^2+^]_i_ recovers memory decline in aged rats

According to above results, we confirmed the correspondence between the degenerations of RBC [Mg^2+^]_i_ and memory performance during aging. However, the causality of whether the metabolism of RBC [Mg^2+^]_i_ could directly influenced the memory function, or if the memory decline caused the RBC [Mg^2+^]_i_ loss, was still unknown. Our following research was aimed at answering this question. Exogenous Mg^2+^ supplement was used to artificially regulate the RBC [Mg^2+^]_i_ level. We were interested in, if this treatment might alter animals’ behavioral performance and if the correlation would still exist.

**Figure 4:**
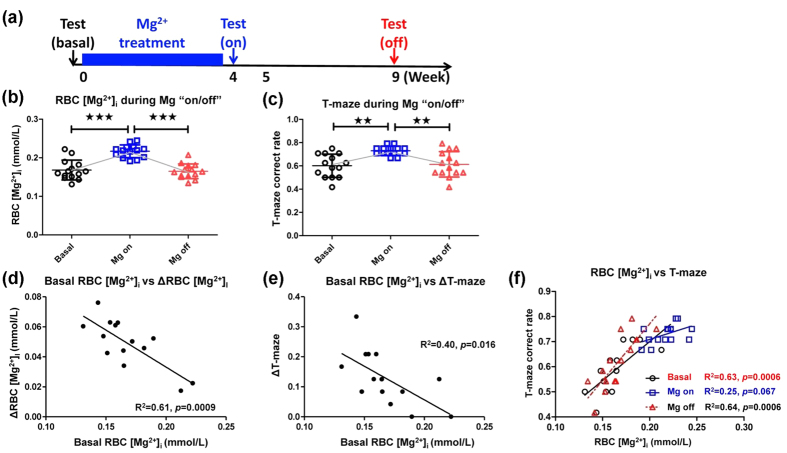
Adjustment of RBC [Mg^2+^]_i_ by exogenous Mg^2+^ supplementation could influence spatial memory. (**a**) Procedure for the “on/off” administration of exogenous Mg^2+^ treatment (aged rats, *n* = 14). (**b,c**) Changes in RBC [Mg^2+^]_i_ and T-maze correct choice rate (8 minutes) during Mg^2+^ “on/off” periods. One-way ANOVA, RBC [Mg^2+^]_i_, F_2,39_ = 27.31, *p* < 0.0001; T-maze, F_2,39_ = 8.94, *p* = 0.0006. ANOVA was followed by Bonferroni’s *post hoc* test, **p < 0.01, ***p < 0.001. (**d**) Inverse linear correlation between basal RBC [Mg^2+^]_i_ and ΔRBC [Mg^2+^]_i_ (RBC [Mg^2+^]_i, on_ − RBC [Mg^2+^]_i, basal_). (**e**) Inverse linear correlation between basal RBC [Mg^2+^]_i_ and ΔT-maze correct choice rate (T-maze_on_ - T-maze_basal_). (**f**) Linear correlation between RBC [Mg^2+^]_i_ and T-maze correct choice rate during Mg^2+^ “on/off”.

A previous study reported that 24 days administration of magnesium-L-threonate (MgT), a highly bioavailable Mg^2+^ compound, could increase the daily Mg^2+^ intake in aged rats, and elevate the CSF [Mg^2+^]^7^. Here, we also used MgT to regulated the RBC [Mg^2+^]_i_ level. Figure 4a shows the procedure of the experiment conducted in the present study. The RBC [Mg^2+^]_i_ and T-maze test were assessed prior to MgT administration (basal level), and then MgT was administered from week 0 to week 4 (Mg^2+^ on). From week 4 to week 5, a secondary measurement of RBC [Mg^2+^]_i_ and a second T-maze test were conducted. This was followed by 4 weeks of normal Mg^2+^ intake to allow the body Mg^2+^ to return to normal level (Mg^2+^ off). At week 9, we did a final correlation test to study the effect of MgT withdrawal.

The exogenous Mg^2+^ treatment successfully improved both RBC [Mg^2+^]_i_ (Fig. 4b) and T-maze correct choice rate (Fig. 4c). Withdrawal of supplementary Mg^2+^ resulted in a return to the basal level for both measurements, so that an “on/off” pattern was evident. Notably, during MgT administration, not all rats shared a similar scale of increases in RBC [Mg^2+^]_i_ or memory, but only the individuals with low basal RBC [Mg^2+^]_i_ levels made remarkable changes. While the smarter rats showed weaker effects of Mg^2+^ administration (as shown in Supplementary Fig. S2). An inverse correlations was evident between the basal value of RBC [Mg^2+^]_i_ and Δ value of RBC [Mg^2+^]_i_/memory (Fig. 4d,e). This suggested a possible threshold for Mg^2+^ therapy: exogenous Mg^2+^ intervention might selectively affect forgetful individuals to a greater extent. The various therapeutic effects of MgT might depend on the different responses of body Mg retention (Supplementary Fig. S3), which would recruit Mg^2+^ in low basal RBC [Mg^2+^]_i_ rats while preventing excessive Mg^2+^ absorbance in normal basal RBC [Mg^2+^]_i_ rats.

Strong intra-group correlations were evident between RBC [Mg^2+^]_i_ and spatial working memory at both the Mg^2+^_basal_ and Mg^2+^_off_ periods (Fig. 4f). The correlation during Mg^2+^_on_ was weak because abundant Mg intake narrowed the deviation among the individuals. In addition, a saturation seemed to show for the effect of Mg^2+^ administration on memory enhancement.

In conclusion, RBC [Mg^2+^]_i_ and memory remained correlated with each other during RBC [Mg^2+^]_i_ regulation, which highlighted the connection between these two items. Elevating the RBC [Mg^2+^]_i_ through exogenous Mg^2+^ administration successfully rescued the memory decline of aged rats, which suggested the subjective effect of body Mg^2+^ status (presented by RBC [Mg^2+^]_i_) to influence animal memory. The memory recovery effect was more pronounced in individuals with lower basal RBC [Mg^2+^]_i_ levels, indicating the function of RBC [Mg^2+^]_i_ to predict therapeutic effect of Mg^2+^ compensation during aging.

### The correlation between RBC [Mg^2+^]_i_ and memory was mediated by brain Mg^2+^ level

**Figure 5:**
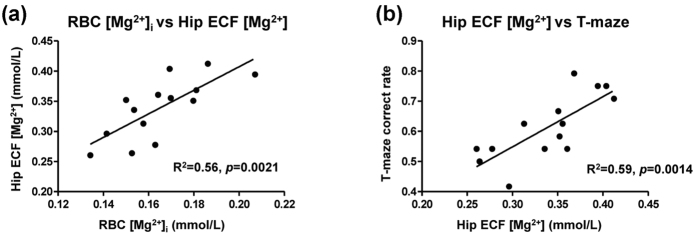
Correlation between RBC [Mg^2+^]_i_ and brain Mg^2+^. (**a**) Linear correlation between RBC [Mg^2+^]_i_ and hippocampus extracellular fluid Mg^2+^ concentration (Hip ECF [Mg^2+^]). (**b**) Linear correlation between Hip ECF [Mg^2+^] and T-maze correct choice rate (data at Mg^2+^ off, Fig. 4).

We next tried to clarify the mechanism underlying the correlation between RBC [Mg^2+^]_i_ and memory. The same group of aged rats was used as in Figs 2~4. Modification of the extracellular [Mg^2+^] was previously reported to affect the function of hippocampal neuron culture^6^. In our present research, the Mg^2+^ concentration in hippocampal extracellular fluid (Hip ECF [Mg^2+^]) was significantly correlated with the RBC [Mg^2+^]_i_ in aged rats (Fig. 5a). The ECF [Mg^2+^], in turn, was significantly correlated with T-maze performance (Fig. 5b).

The correlation between RBC [Mg^2+^]_i_ and CSF [Mg^2+^] was not significant in aged rats (Supplementary Fig. S4a); however, dividing the rats into normal and low groups, according to their RBC [Mg^2+^]_i_ values (dotted line in Supplementary Fig. S4a), revealed a significant intergroup difference in the CSF [Mg^2+^] level (Supplementary Fig. S4b, right).

These data revealed a link between peripheral Mg^2+^ and CNS Mg^2+^ (also shown in Fig. 1c). An exchange of Mg^2+^ apparently occurred between the brain and periphery through the whole body blood circulation. The metabolism of body Mg^2+^ pool would therefore affect both the ECF [Mg^2+^] and RBC [Mg^2+^]_i_, thereby contributing to the observed correlation. Although the Hip ECF [Mg^2+^] could more accurately represent the Mg^2+^ status in the functional brain region, the RBC [Mg^2+^]_i_ is much easier to obtain and safer to assess.

### Correlations among RBC [Mg^2+^]_i_, Hip ECF [Mg^2+^], synapse density, and memory

The synapse is the basic unit for building the neural network. Previous work showed that synaptic density in the hippocampus, which showed an increase by Mg^2+^ oral administration, was significantly correlated with memory performance^7^. Therefore, we examined whether the RBC [Mg^2+^]_i_, which indicated the memory function, would also be correlated with the synapse density in aged rats.

**Figure 6:**
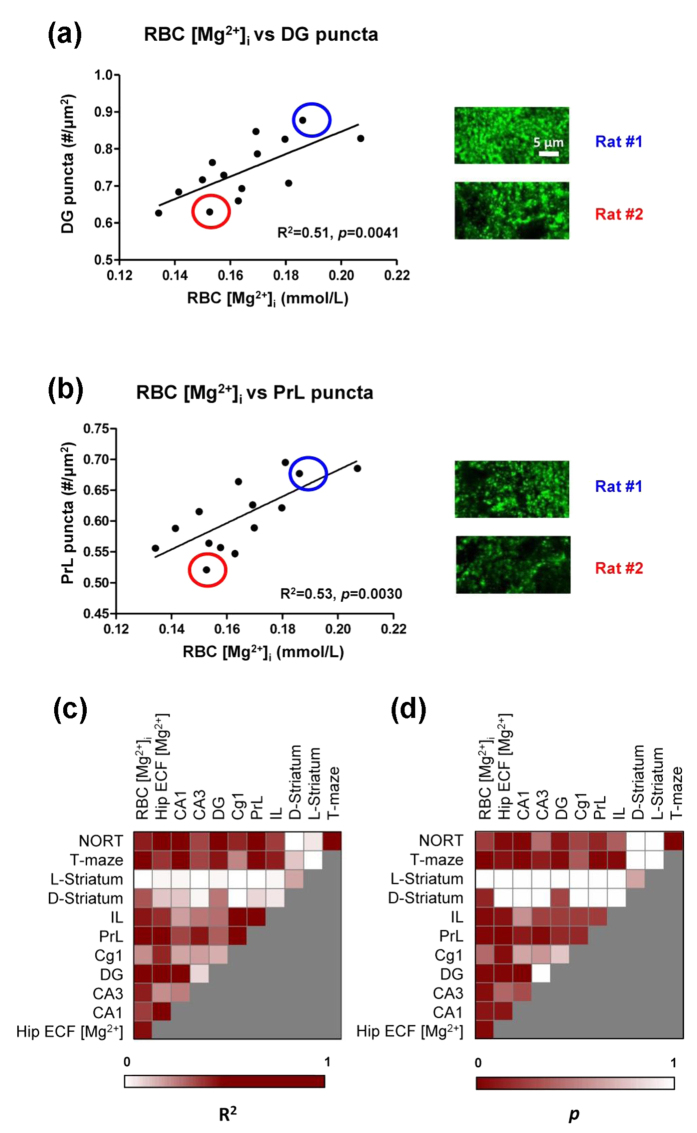
Correlation between RBC [Mg^2+^]_i_, Hip ECF [Mg^2+^] and Syn-(+) punctal density. (**a**) Linear correlation between the RBC [Mg^2+^]_i_ and Syn-(+) puncta density in dentate gyrus (DG) subregion of Hip (aged rats, *n* = 14). The density was estimated as the number of immunostained puncta per μm^2^ (#/μm^2^). Rat #1 (blue circle) and rat #2 (red circle) were selected; their immunostaining images were showed as examples. (**b**) Linear correlation between the RBC [Mg^2+^]_i_ and Syn-(+) puncta density in the PrL subregion of the prefrontal cortex (PFC). The immunostaining images of rat #1 and rat #2 were showed as examples. (**c**,**d**) Heat maps of linear analysis for [Mg^2+^], Syn-(+) puncta density, and memory (c, R^2^; d, *p*).

Immunostaining of synaptophysin was used to identify presynaptic terminus. The density of synaptophysin-positive (Syn-(+)) puncta was used to evaluate synapse density. A strong correlation between RBC [Mg^2+^]_i_ and Syn-(+) puncta density was found in the dentate gyrus (DG) subregion of Hip (Fig. 6a), as well as in the PrL subregion of medial prefrontal cortex (mPFC, Fig. 6b). Figure 6c,d show the statistical heat maps of the linear correlation analysis for all the items, arranged in a logical sequence from peripheral Mg^2+^ (RBC [Mg^2+^]_i_), brain Mg^2+^ (Hip ECF [Mg^2+^]), and synapse density (8 different brain subregions), to memory performance (NORT and T-maze). The dark brown squares indicate the strongest correlations. Synapse densities in the dorsal and lateral striatum were used as controls, which showed no significant correlation with almost any of the other items.

## Discussion

In the present work, for the first time, we found that endogenous Mg^2+^ level, which was represented by the RBC [Mg^2+^]_i_, was correlated with recognition memory and spatial memory in rats. The possible mechanism for this phenomenon was: the RBC [Mg^2+^]_i_ represented the brain ECF [Mg^2+^] level, the latter affected the density of Syn-(+) puncta and ultimately regulated memory. We also confirmed the aging associated endogenous Mg^2+^ deficiency, and found that compensation of RBC [Mg^2+^]_i_ deficiency by exogenous Mg^2+^ administration rescued the memory decline in aged rats. The Mg^2+^ administration selectively improved the memory of individuals with low basal RBC [Mg^2+^]_i_, showing an inverse correlation between basal RBC [Mg^2+^]_i_ level and therapeutic effect. Our original technique for RBC [Mg^2+^]_i_ measurement may provide a potential tool for clinical diagnosis and treatment of aging-associated memory decline.

Before the correlation studies, we established a screening protocol to provide an ideal Mg^2+^ index to indicate body Mg^2+^ status. Our focus on the blood Mg^2+^ pool has many advantages. First, blood samples are very easy to collect, causing little hurt to animals, allowing real time and repeated assays. Second, blood regulates the Mg^2+^ content in tissues of the whole body through Mg^2+^ exchange, which raises the possibility that blood [Mg^2+^] could represent the Mg^2+^ levels in other tissues. Third, the role of blood Mg^2+^ as a biomarker in peripheral diseases is well established. Some reports have already shown significant changes in blood Mg^2+^ pools in hypertension and diabetes patients (Supplementary Table S2).

Blood Mg^2+^ exists in different compartments, which consists of plasma, erythrocytes, leukocytes, and thrombocytes. Plasma [Mg^2+^], the extracellular Mg^2+^ pool, was not sufficient to represent Mg^2+^ decrease in metabolic syndrome (Supplementary Table S2), and failed to reflect brain [Mg^2+^] level^34^,^35^. Among the intracellular Mg^2+^ compartments, erythrocytes make up more than 90% of the total blood cells, therefore mainly affect the intracellular blood Mg^2+^ content. Within erythrocytes, 90% of the Mg^2+^ binds to biomolecules, leaving about 10% in ionized form. This free Mg^2+^ ([Mg^2+^]_i_) is the real Mg^2+^ component with the most bioactivity, which regulates intracellular physiological reactions and exchanges Mg^2+^ content with extracellular plasma pool. Thus, the [Mg^2+^]_i_ of erythrocytes might represent the Mg^2+^ status of blood cells and might further correlate with the body Mg^2+^ status.

It has been previously reported that RBC [Mg^2+^]_i_ corresponded to other blood Mg^2+^ pools during body Mg^2+^ decline in the processing of hypertension/diabetes^8^,^9^,^10^,^11^,^12^. In our studies, the RBC [Mg^2+^]_i_ not only correlated to the Mg^2+^ levels in peripheral tissues, but also correlated to brain ECF [Mg^2+^]. A further correlation between RBC [Mg^2+^]_i_ and memory was found, which still persisted when RBC [Mg^2+^]_i_ was adjusted by Mg^2+^ intake “on/off”. Our findings redefined the biomarker function of RBC [Mg^2+^]_i_: not only being a Mg^2+^ marker in the periphery, but also being a memory indicator in the CNS.

Synapse is the elementary unit to build the neural network. The number of synapses determines the computational capability of a neural system, further regulates the memory function. Animal and human studies have shown strong correlations between synapse density and memory performance during normal aging^36^,^37^,^38^. Our previous research demonstrated that chronic Mg^2+^ treatment enhanced synapse density as well as memory functions in aged rats^7^. One of the crucial Mg^2+^ functions to regulate synapse density is through interaction with NMDAR. In brain, Mg^2+^ binds to NMDAR as a voltage-gated regulator. It inhibits background NMDAR transmission and Ca^2+^ influx, without further blocking burst input^7^. Frequent background Ca^2+^ influx may activate some neural inhibitory factors (calcineurin, e.g.) and downregulate NMDAR transmission^39^,^40^. While chronic suppression of background Ca^2+^ influx by physiological level Mg^2+^ (burst stimulus was not inhibited) could lead to: enhancement of NMDAR (especially NR2B subunit) signaling and CaMKII/CREB/BDNF pathway, increasing of synaptic protein expression, elevation of synapse density and reconfiguration of synaptic networks^6^,^7^,^41^. Meanwhile, in the aspect of energy supply, intracellular Mg^2+^ could regulate the function of mitochondria and increase ATP, thus also facilitates the expression and trafficking of synaptic protein^42^.

During aging, Mg^2+^ deficiency may attenuate its prevention function from over-excitation of NMDAR by background input, which would result in cascaded negative effect as: persistent increase of intracellular Ca^2+^ level, abnormalities of Ca^2+^-related signal pathway and mitochondria function, decreasing of synaptic protein expression, decline of synaptic plasticity and synapse density, and finally deficiency of cognitive function^43^,^44^,^45^,^46^.

Although above evidences suggested a possible function of brain Mg^2+^ to regulate synapse density *in vivo*, no previous analysis of endogenous Mg^2+^ level could support this hypothesis. In the present work, we have provided the first quantitative analysis of the relationship between brain Mg^2+^ level and the synapse density in the hippocampus (Fig. 6c,d). The Hip ECF [Mg^2+^] was significantly correlated with the density of Syn-(+) puncta in different subregions of the Hip, with the strongest correlation found in the DG. The synapse density in the DG was further correlated with the recognition and spatial memory, in agreement with earlier work^7^,^38^.

The mPFC also plays an important role in learning and memory. The synapse density in the PrL subregion was the most strongly correlated with memory. The striatum was reported to influence the transformation of space/response acquisition strategy in the T-maze test^47^, which was not found in our experiment. So striatum was used as a control in the present work. We found no correlations between synapse density of the striatum and the T-maze score (Fig. 6c,d).

**Figure 7:**
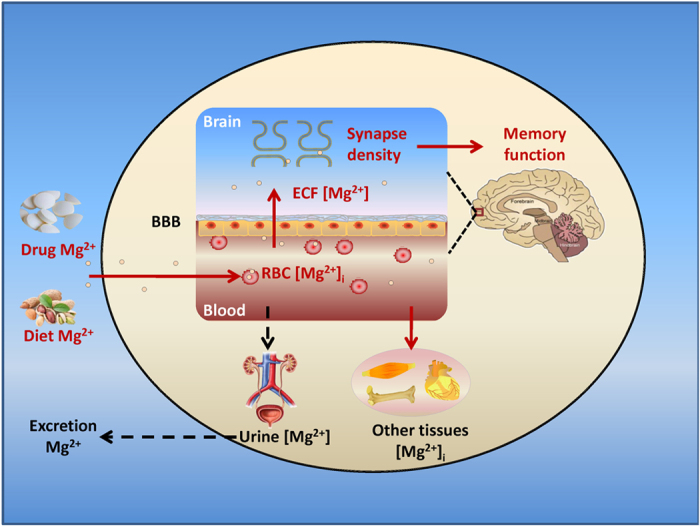
Model of the correlation among periphery Mg^2+^, brain Mg^2+^, synapse density, and memory. In the normal condition, RBC [Mg^2+^]_i_ is regulated by exogenous dietary Mg^2+^ intake and urine Mg^2+^ excretion. RBC [Mg^2+^]_i_ represents the status of blood Mg^2+^. Mg^2+^ is exchanged between the blood and other tissue in periphery and between the blood and brain ECF through the blood brain barrier (BBB). The brain ECF [Mg^2+^] regulates synapse density in the Hip and PFC, further modulating memory function. During aging, abnormal metabolism of Mg^2+^ between the body and the external environment leads to a decline in RBC [Mg^2+^]_i_, which influences ECF [Mg^2+^] and further impairs memory. Enhancing Mg^2+^ intake through diet and/or drugs could affect the metabolic balance of body Mg^2+^ and rescue the degeneration of memory function.

With above findings, we have constructed a logical model (Fig. 7). The RBC [Mg^2+^]_i_, ECF [Mg^2+^], synapse density, and memory are correlated with each other. The blood and brain ECF exchange Mg^2+^ through the BBB. While the brain ECF [Mg^2+^] regulates synapse density in the Hip and mPFC functional regions, further modulating recognition and spatial memory functions. During aging, a degenerated Mg^2+^ metabolism from the periphery to the brain leads to downregulation of synapse density and impairment of memory. Exogenous administration of Mg^2+^ could rescue these symptoms. The utilization of RBC [Mg^2+^]_i_ assay provides an easy, repeatable, and representative technique in the fundamental research for studying the role of Mg^2+^ in neural activity and memory function.

The exogenous Mg^2+^ supplementation experiments in aged rats showed that RBC [Mg^2+^]_i_ and memory corresponded with each other during the Mg^2+^ “on/off” periods, which highlighted the correlation between these two items. Modification of the body Mg^2+^ status directly affected the behavioral score, which suggested not only the associated relationship, but also the subjective role of the endogenous Mg^2+^ level to influence memory. This finding may help in clinical studies of the Mg^2+^ therapy for preventing endogenous Mg^2+^-deficiency-associated memory decline during aging.

Interestingly, we noticed that elevating the Mg^2+^ intake sufficiently rescued the memory decline in individuals with low basal RBC [Mg^2+^]_i_, whereas aged rats with normal baseline RBC [Mg^2+^]_i_ levels were not affected. This finding reflects two facts. The first is, not all aged animals have endogenous Mg^2+^ losses and memory decline. Aging is not stringently corresponding to the degeneration of memory function, but only increases the risk; while RBC [Mg^2+^]_i_, at least on our experimental platform, shows a strict correspondence with memory performance, making itself a more precise memory indicator. From this perspective, in the studies of aging associated memory decline, it is not enough to define “aging” with only “how many years old the subject is”; but a differentiation of the individual RBC [Mg^2+^]_i_ levels is also required. Using “Mg^2+^ age” to delimit “aging” and memory deficit is more representative (Supplementary Fig. S5). Secondly, the phenomenon of Mg^2+^ supplement “on/off” also suggests a characteristic of the Mg^2+^ medicinal value: Mg^2+^ treatment may selectively recover the memory of forgetful individuals to a normal level, without further influencing normal individuals to be “smarter”, according to their RBC [Mg^2+^]_i_ levels. This reveals the function of “Mg^2+^ age” measurement to conduct the precise medication for memory decline subjects. There is an inverse correlation between the therapeutic memory improvement and basal RBC [Mg^2+^]_i_. Old individuals with normal “Mg^2+^ age” may not need Mg^2+^ compensation, while patients with low “Mg^2+^ age” do.

Aging-associated Mg^2+^ declines have already been revealed in humans^8^,^48^. Insufficient Mg^2+^ intake might lead to more serious Mg^2+^ loss and faster impairment of memory in aging people. Some surveys indicated the lack of Mg^2+^ intake in a significant portion of the population in industrialized countries. For example, more than 60% of Americans did not meet the RDA-DRI criteria for daily Mg^2+^ intake^49^; while average Mg^2+^ intake in the aging population declined to one half of the RDA^14^. RBC [Mg^2+^]_i_, which accurately represents body Mg^2+^ status, might therefore serve as an ideal clinical biomarker for Mg^2+^ deficiency diagnosis and Mg^2+^ compensation therapies. The distinction between aged people with normal “Mg^2+^ age” and pathological “Mg^2+^ age” allows individualized Mg^2+^ administration in the clinical setting. As looking forward, our research may provide an easy and feasible therapeutic solution for precision medicine of endogenous Mg^2+^-deficiency-induced memory decline in aging humans.

## Methods and Materials

### Experimental animals

Male Sprague-Dawley rats were obtained from Vital River Laboratory (Animal Technology Co. Ltd., Beijing, China). All animals were housed individually, with free access to standard food and water, in a controlled environment (temperature 21 ± 1 °C, humidity 50 ± 10%), under a 12:12 hour inverted light-dark cycle (light off from 8:00 A.M. to 8:00 P.M.). On arrival and during the whole experiment process, rats were fed a commercial pelleted diet (SLAC Laboratory Animal Co. Ltd, Shanghai, China), containing a normal Mg^2+^ concentration (0.15%). For Mg^2+^ intake deficiency experiment, 3-month-old rats were used (n = 10). For other experiments, young (3 month) and aged (22 month) rats were used (n = 14/group).

All experiments involving animals were approved by the Tsinghua University committees on animal care. All the methods were carried out in accordance with the approved guidelines and regulations.

### Mg^2+^ intake deficiency

The Mg^2+^ deficient diet (Chinese Academy of Agricultural Sciences, Beijing, China) was composed of 180 g/kg casein, 100 g/kg sucrose, 588 g/kg cornstarch, 30 g/kg α-cellulose, 50 g/kg soybean oil, 2 g/kg methionine, 40 g/kg mineral mix, and 10 g/kg vitamin mix. The total elemental Mg^2+^ concentration in the Mg^2+^ deficient food was 0.003% (compared to 0.15% in standard diet), as determined by inductively coupled plasma atomic emission spectrometry (ICP-AES, Chinese Academy of Agricultural Sciences).

For the Mg^2+^ intake deficiency test, two groups of rats were fed with either standard food or Mg^2+^ deficient food for 4 weeks. Rat chow was weighed to calculate the daily total Mg intake. A body Mg retention test was also assessed during this period (see Supplementary Information). At the end of week 4, blood samples were taken for RBC [Mg^2+^]_i_ determinations, then all animals were sacrificed, the tissues, and skeletons were taken for total Mg measurements, CSFs were taken for CSF [Mg^2+^] survey.

### Mg^2+^ measurement

We designed an original technique for RBC [Mg^2+^]_i_ determination. Blood was taken from the caudal vein of rat and transferred into heparin coated tube. The whole blood sample was diluted 1:1200 in Hanks Balanced Salts (HBSS, without Ca^2+^ and Mg^2+^, Sigma-Aldrich) containing 0.5 mmol/L Mg^2+^. A cell-permeable Mg^2+^ dye, Magnesium Green (MaG, ex_max_  =  506 nm, em_max_ = 531 nm, Invitrogen), was used to detect RBC intracellular free Mg^2+^, with a working concentration of 5 mg/L. Pluronic F-127 (Invitrogen) was used to aid dissolution of MaG. The mixture of RBCs and MaG was incubated at 37 °C for 60 minutes, washed twice, and incubated at 37 °C for another 30 minutes. The fluorescence signal was detected by flow cytometry (BD FACSCalibur). The erythrocyte population was gated by forward scatter (FSC)/side scatter (SSC) dotted diagram. A total of 1 × 10^4^ erythrocytes in FL1 were selected for average fluorescence intensity (Supplementary Fig. S6). For calibration, parallel samples were washed twice with HBSS (zero Mg^2+^) and then mixed in HBSS with a gradient of [Mg^2+^]. A23187 (Sigma-Aldrich) was used to maintain the external and internal [Mg^2+^] balance across the cellular membrane, with a working concentration of 25 μmol/L. Mixtures were incubated at 37 °C for 2 hours and then analyzed by flow cytometry. A fitted curve was built using fluorescence intensity as *x*-axis and standard [Mg^2+^] gradient as *y*-axis (Supplementary Fig. S7a). Similar as [Mg^2+^]_i_ calibration, the influence of different [Ca^2+^]_i_ (with fixed [Mg^2+^]_i_ = 0.25mM) to MaG fluorescence was also evaluated (Supplementary Fig. S7b). There was no influence of [Ca^2+^] change at basal level.

For tissular total Mg determination, the rats were anesthetized, and sacrificed by decollation. The hearts, kidneys, muscles, livers, brains, intestines, and femurs were harvested, dried and weighed. The dry samples were analyzed using inductively coupled plasma mass spectrometry (ICP-MS, General Research Institute for Nonferrous Metals, Beijing, China). Data were analyzed by dividing the tissular total Mg of each rat in low Mg diet group with average tissular total Mg in normal Mg diet group, as below:


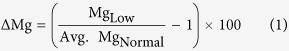


For determination of CSF [Mg^2+^], rats were anesthetized with chloral hydrate (300 mg/kg, i.p.) and CSF was collected from the cisterna magna using a 1 mL syringe. The Mg^2+^ level in CSF was determined by calmagite chromometry^50^.

For determination of extracellular fluid (ECF) [Mg^2+^], rats were anesthetized with chloral hydrate (300 mg/kg, i.p.) and positioned onto a stereotaxic apparatus. A midline incision of the skull was executed and a small hole was made in the skull using a dental driller. A microdialysis guide cannula (CMA) was implanted into hippocampus (AP −5.2 mm, L −3.0 mm, V −4.2 mm). The guide cannula was fixed with light-solid dental cement. The rats were allowed to recover for 1 week before *in vivo* microdialysis sampling. During sampling, a microdialysis probe (CMA, dialysis length, 2 mm; OD, 0.5 mm) was inserted into the guide cannula in anesthetized rat. Artificial CSF solution (aCSF) was perfused at a flow rate of 0.2 μL/minute, for at least 30 minutes for equilibration. Samples were continuously collected for 2 hours on ice. The Mg^2+^ level in the ECF was determined by calmagite chromometry.

### Novel object recognition test

The novel object recognition test (NORT) was used to evaluate the recognition memory of young and aged rats. The apparatus consisted of a square arena (60 × 60 × 40 cm) constructed from polyvinyl chloride, with black walls and floor. An overhead camera and a video recorder were used to monitor and record the animal’s behavior for subsequent analysis. The protocol of NORT was optimized to expand the dispersion of individuals for a better correlation study, while it could still distinguish the difference between young and aged animals (see Results 3.2).

Two days before the experiment, rats received two sessions of habituation to the arena and test room for 10 minutes/session/day. On the third day, each rat was placed in the box and exposed to 3 different objects for 5 minutes (sample phase), and then returned to its cage. The box and objects were cleaned between trials to prevent the buildup of olfactory cues. The number of times that the rats explored each object was then counted. Twenty-four hours later, the object with the maximum number of counts, which differed for each rat, was replaced with a novel object. The rat was then placed back in the box for another 5 minutes (acquisition phase). The recognition index was calculated as the percentage ratio of counts on the novel object over the total counts during the acquisition phase. There were no differences in the motor abilities, explorative abilities and object preference (rats explore one object much more/less than the other objects in the sample phase) between young and aged rats (see Supplementary Fig. S8).

### T-maze test

Spatial working memory was assessed using a T-maze task in young and aged rats. Briefly, rats were maintained on a restricted feeding schedule at 85% of their free-feeding weight. The maze was located 1 m above the floor, in a dark room with extra-maze cues. The rats were handled and habituated to the maze for 10 days. Each trial consisted of a sample run and a choice run, with a delay interval of 15 seconds during the training. For the sample run, two patches of chocolate were placed on each arm of the maze. The rat was forced to enter the left or right arm randomly, by the presence of a block. After a delay interval, on the choice run, the block was removed and the rat was allowed a free choice of either arm. The animal was rewarded by the other chocolate for choosing the opposite arm it had visited before. This was called a correct choice. Rats were run one trial at a time, with an inter-trial interval of 20 minutes. Each daily session consisted of 8 trials. At the end of the training sessions, all animals had reached a >90% correct choice level.

The testing sessions were the same as training sessions, except that the delay interval time was extended from 15 seconds to 4 or 8 minutes. For correlation analysis, the 8 minute delay test was repeated for 3 consecutive days, with a total of 24 trials for each rat. Rats used space rather than response acquisition strategy when the maze was rotated 180° (data not shown). After the T-maze test, blood samples were taken to determine RBC [Mg^2+^]_i_.

In the Mg^2+^ treatment procedure, MgT was administered via the drinking water (50 mg/kg/day elemental Mg^2+^). This Mg^2+^ compound was highly bio-affinitive and successfully increased the Mg^2+^ in rodent brain via dietary supplementation^7^.

### Brain preparation and fluorescent immunostaining

The procedures for brain perfusion, brain fixing, slice cutting, and immunostaining were described before^7^. Briefly, rats were anesthetized with chloral hydrate, perfused with phosphate buffer saline (PBS), and fixed with 4% paraformaldehyde. Coronal sections (5 μm) from the hippocampus, prefrontal cortex, and striatum were cut on a cryostat (Leica). For immunostaining, the semi-brain slices were blocked in PBS containing 3% rabbit serum (Gibco) and 0.2% TritonX-100. Sections were incubated with mouse anti-synaptophysin antibody (1:500, Millipore) and Alexa 488-coupled rabbit-anti-mouse IgG (1:200, Invitrogen). Mounting medium (Vector Laboratories) was used to prevent fluorescence fading.

### Estimation of synaptophysin-positive puncta density

The synapse density could be estimated by immunostaining of synaptophysin (Syn), which binds to the vesicles at the presynaptic release site, and participates in synaptic transmission. Measurement of Syn-(+) puncta can be used for the quantification of synapses. The procedures for confocal scanning and signal processing were described previously^7^. Briefly, stained brain sections were imaged with an Olympus IX-70 confocal microscope with a 60× water lens at zoom ×3, generating an image of 78.6 × 78.6 μm dimensions. Serial z-sectioning was performed (0.6 μm thick) and the best three z-sections (with the highest number of puncta) were collected and merged into a single image. Therefore, the volume of brain tissue per image was 78.6 × 78.6 × 1.8 μm^3^. The density of synaptophysin-positive puncta was estimated from the obtained images using Image-Pro-Plus software version 6.0 (Media-Cybernetics). Background levels were equalized and special filters were applied to separate fluorescent puncta. Settings for each image were adjusted to maximize the number of detected fluorescent puncta. The mean punctum number/μm^2^ was used as an estimate of the presynaptic punctal density.

### Statistics

Data are presented as mean ± SD. All data comparing two groups were analyzed using unpaired *t* tests. Correlations were analyzed using linear regression. The T-maze retention interval curve was analyzed using two-way ANOVA, followed by Bonferroni’s post hoc test. The Mg^2+^ “on/off” experiment was analyzed using one-way ANOVA, followed by Bonferroni’s post hoc test. Statistical significance was defined as *p* < 0.05.

## Additional Information

**How to cite this article**: Xiong, W. *et al*. Erythrocyte intracellular Mg^2+^ concentration as an index of recognition and memory. *Sci. Rep.* **6**, 26975; doi: 10.1038/srep26975 (2016).

## Supplementary Material

Supplementary Information
