# Supplementary material for: Erythrocyte intracellular Mg2+ concentration as an index of recognition and memory
Source: Sci Rep. 2016 Jun 2;6:26975. doi: 10.1038/srep26975 (PMC4890594; doi:10.1038/srep26975)
Supplement: Supplementary Information [file srep26975-s1.pdf]

Erythrocyte intracellular Mg^{2+} concentration as an index of recognition and memory

Wenxiang XIONG, Yaru LIANG, Xue LI, Guosong LIU, Zhao WANG*

MOE Key Laboratory of Protein Sciences, Department of Pharmacology, School of Medicine, Tsinghua University, Beijing 100084, P.R. China

* Correspondence and requests for materials should be addressed to Dr. Zhao WANG at Department of Pharmacology, School of Medicine, Tsinghua University, Beijing 100084, P.R. China.

E-mail: zwang@tsinghua.edu.cn

Phone: +86 10 6277 2240; Fax: +86 10 6277 2675

Supplementary information

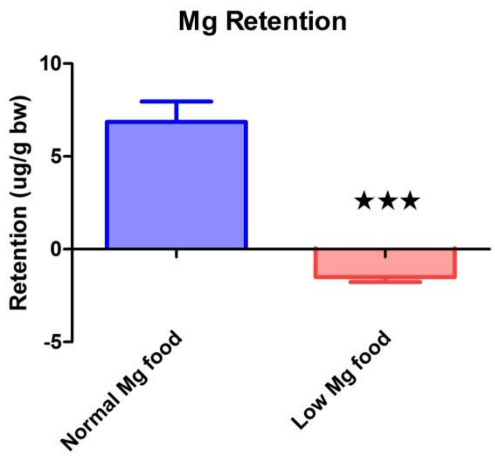

Supplementary Figure S1: Mg^{2+} intake deficiency induced a decrease in body Mg retention. The Mg retention (method see below) in low Mg^{2+} diet group dramatically dropped from positive to a negative value, indicating a Mg^{2+} loss from body.

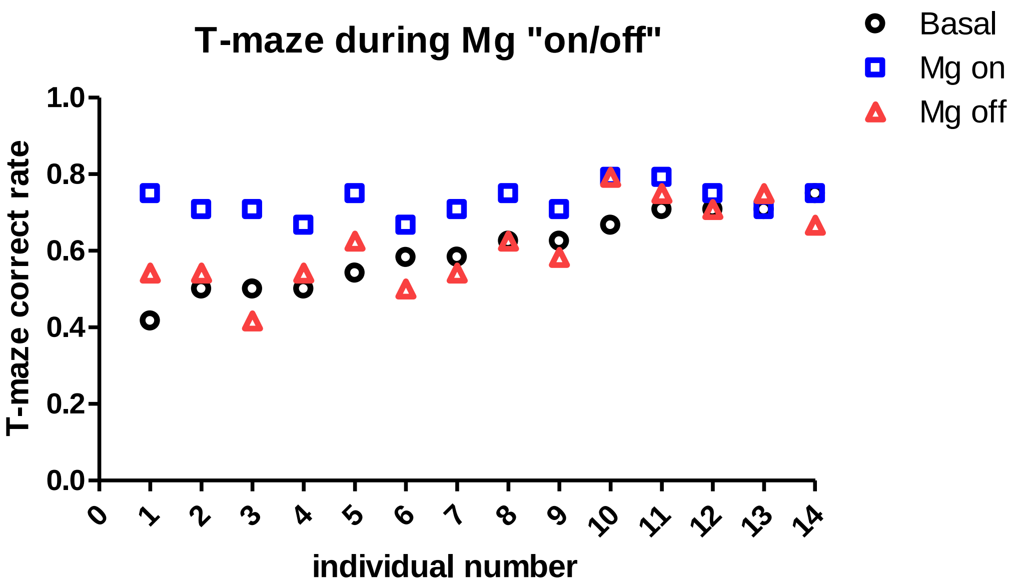

Supplementary Figure S2: Administration of MgT showed less effect on aged rats with higher basal memory status. The x-Axis represents individual rat numbers, which were arranged in ascending order based on their basal values (black).

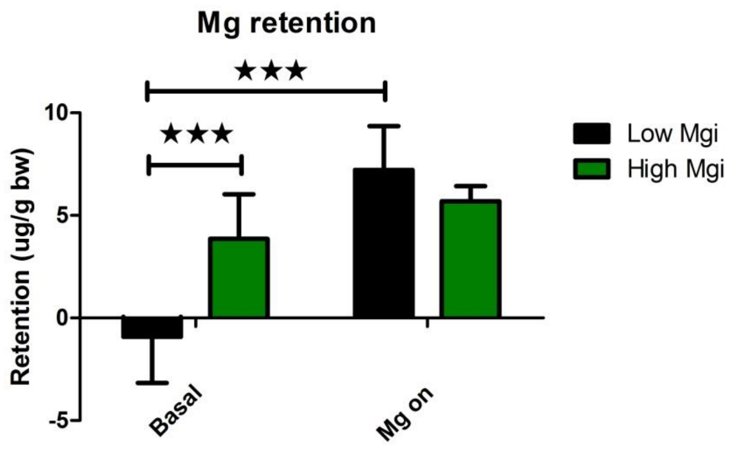

Supplementary Figure S3: Effect of MgT treatment on body Mg retention. Rats were divided into low and normal groups according to their basal RBC $[Mg^{2+}]_i$ values**.** During MgT treatment, RBC $[Mg^{2+}]_i$ was regulated by body Mg retention efficiency. Only Mg retention in the low RBC $[Mg^{2+}]_i$ group showed strong response to MgT treatment. Data are presented as mean ± SD. Two-way ANOVA was followed by Bonferroni's *post hoc* test, ****p*<0.001.

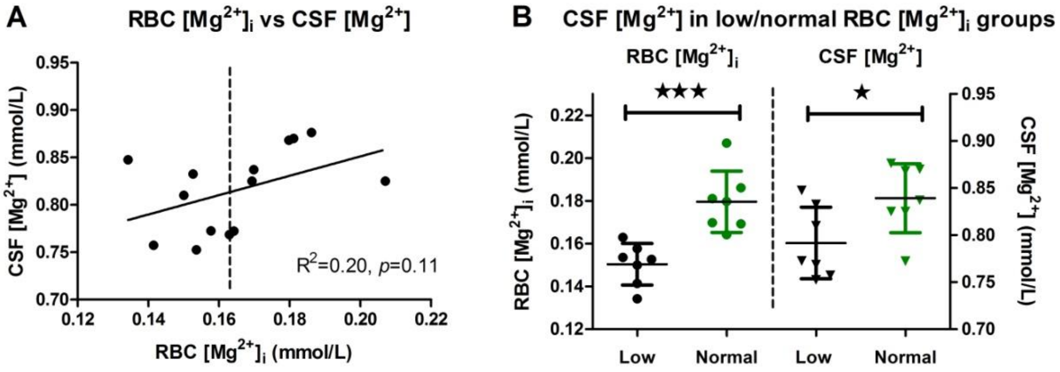

Supplementary Figure S4: Correlation between RBC $[Mg^{2+}]_i$ and CSF $[Mg^{2+}]$. (A) Linear correlation analysis between RBC $[Mg^{2+}]_i$ and CSF $[Mg^{2+}]$ in aged rats (*n*=14) (dotted line, rats were divided into normal and low groups according to their RBC $[Mg^{2+}]_i$ values). (B) Comparison of CSF $[Mg^{2+}]$ based on individual RBC $[Mg^{2+}]_i$ levels. Unpaired *t* test, *$p<0.05$, ***$p<0.001$.

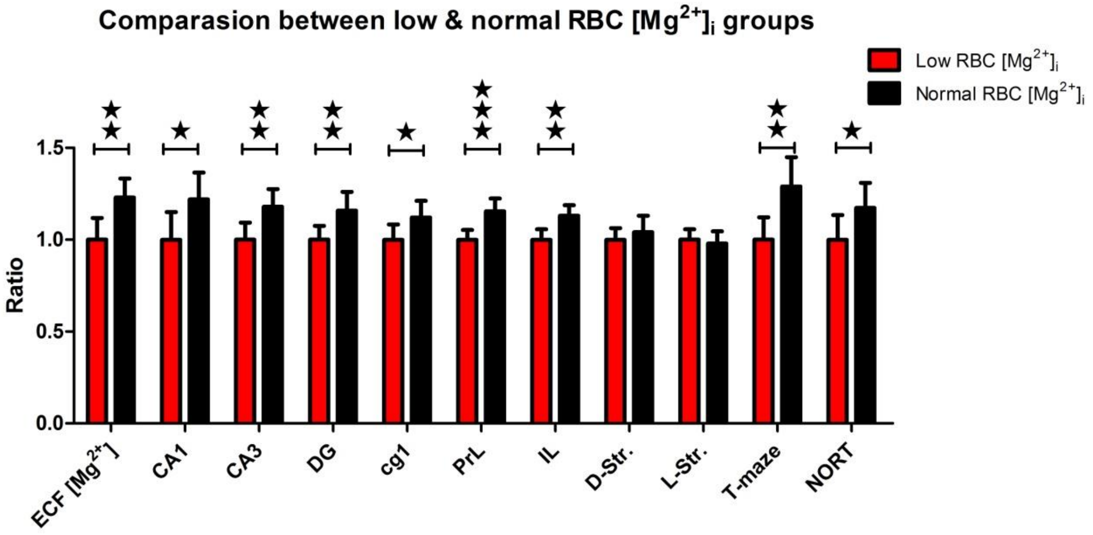

Supplementary Figure S5: Differentiation of [Mg^{2+}], synapse density, and memory levels based on individual RBC [Mg^{2+}]$_i$ values. Significant differences between low RBC [Mg^{2+}]$_i$ group and normal RBC [Mg^{2+}]$_i$ group were shown. Data are presented as mean ± SD. Unpaired t test, **p*<0.05, ***p*<0.01, ****p*<0.001.

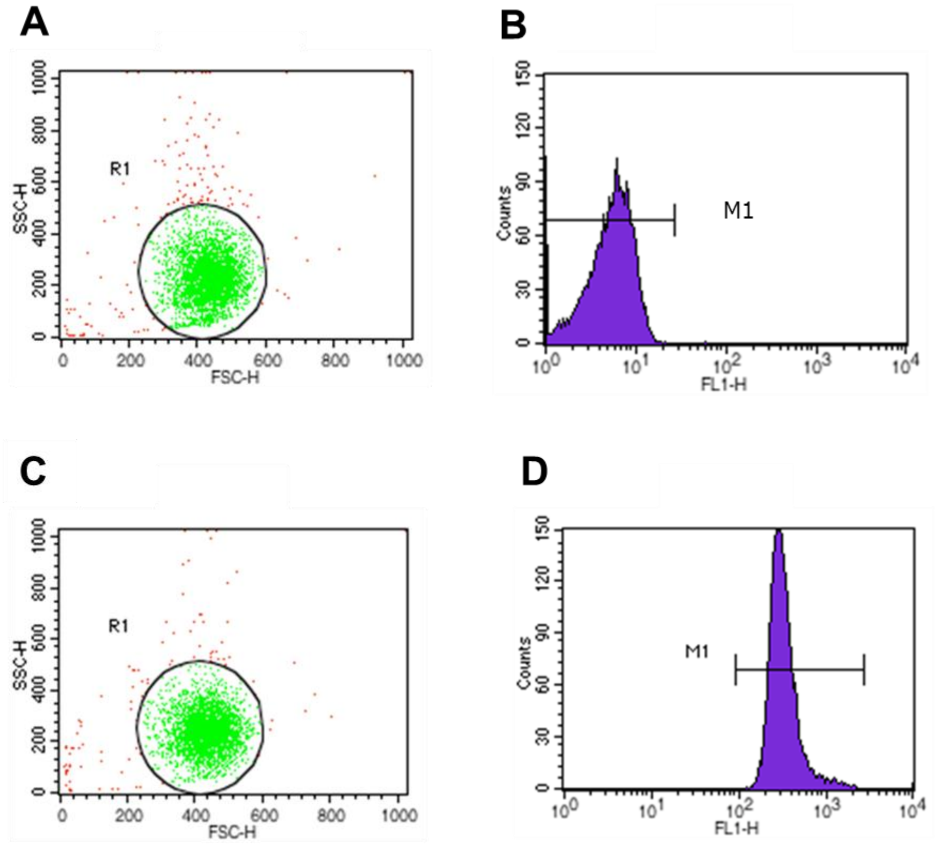

Supplementary Figure S6: The flow cytometry images of Mag staining in erythrocyte. (A) The forward scatter (FSC)/side scatter (SSC) image of an erythrocyte sample incubated without Mag dye (control). (B) The FL1/counts image of the control. (C) and (D), the FSC/SSC and FL1/counts images of the same erythrocyte sample incubated with Mag dye loading.

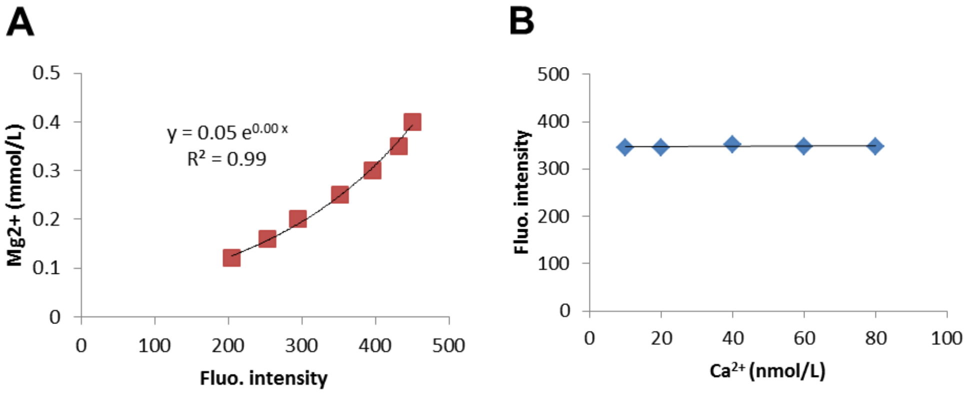

Supplementary Figure S7: The calibration of MaG fluorescence intensity. (A) A fitted curve was built using fluorescence intensity as x-axis and standard [Mg^{2+}] gradient as y-axis. The real RBC [Mg^{2+}]$_i$ in each sample was calculated by inputting the fluorescence intensity value to the fitting formula. (B) The influence of [Ca^{2+}]$_i$ changes to MaG fluorescence was evaluated by incubating the erythrocytes with [Ca^{2+}] gradient (similar as [Mg^{2+}]$_i$ calibration, with fixed [Mg^{2+}]$_i$ = 0.25mM) and comparing the related fluorescence. There was not Ca^{2+} influence in erythrocyte at physiological level.

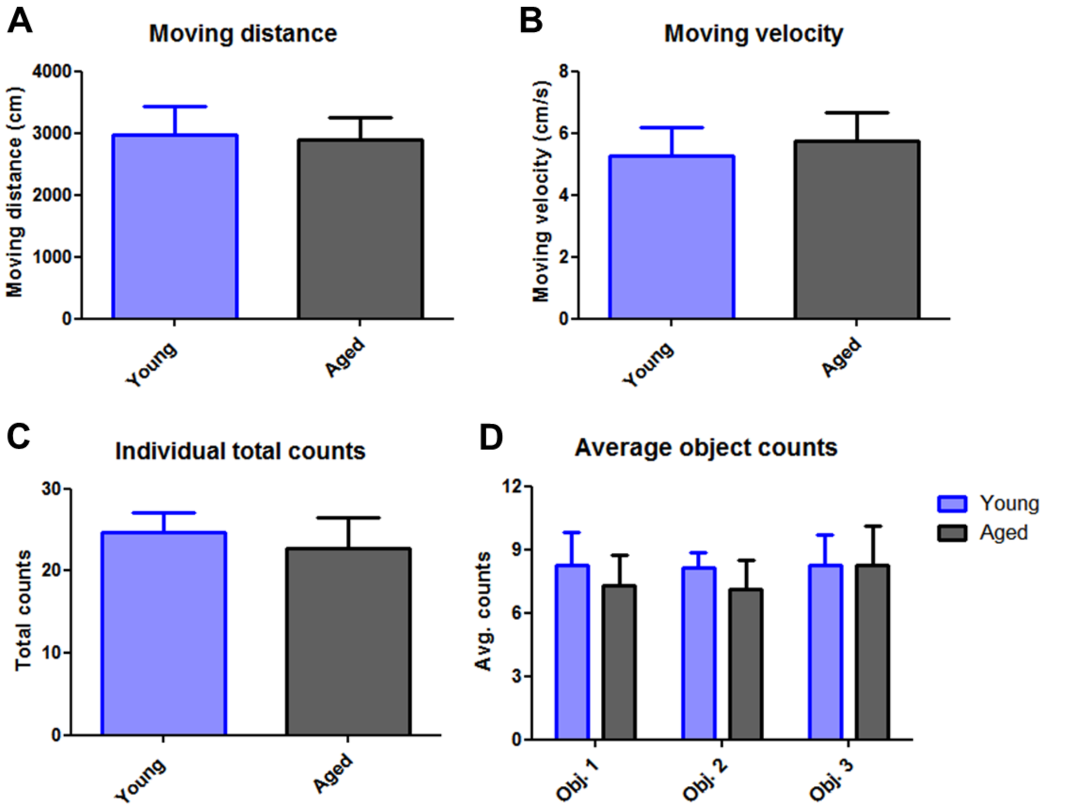

Supplementary Figure S8: Rats did not show age related deficits in motor abilities, explorative abilities and object preference. The total moving distance (A) and velocity (B) were measured in the habituation phase. There were no differences between young and aged rats (the same rats as Fig. 2~6; n=7/group). (C) In the sample phase, the total counts of all the 3 objects for each rat were calculated. There were no differences between young and aged rats, suggesting similar explorative abilities between the 2 groups. (D) Also in the sample phase, the number of counts for each object was compared. There were not significant differences among the average counts of the 3 objects, both in young and aged groups. It suggested that there was not an object preference which may interfere the experiment (rats explore one object much more/less than the other objects in the sample phase).

Mg retention procedure:

To assess Mg retention, animals were individually housed in metabolic cages. Animals received either normal food or Mg deficient food, with either deionized water or water containing MgT. After 3 days of habituation, urine and fecal pellets from each rat were collected on a daily basis over a continuous five-day-period. Food in each cage was weighed every day to calculate the daily Mg intake. Rats were weighed. The urine and fecal pellets were analyzed for Mg content using ICP-AES. The percentage of retention was estimated using the following equation:

$$\text{Mg Retention} = \frac{\text{Mg}_{\text{intake}} - \text{Mg}_{\text{feces}} - \text{Mg}_{\text{urine}}}{\text{Body weight}}$$ Supplementary Equation S (1)

Supplementary Table S1: Mg detection in tissues

Research field	Species	Assay tech.*	Liver	Kidney	Heart	Muscle	Muscle ionized	Brain	Brain ionized	Skeleton	Intestine	Reference
Alzheimer's Disease	Human	ICP-AES						√				1
Congestive heart failure	Human	AAS			×	×						2
Genetic low Mg, Mg deficient food	Mouse	AAS		√						√		3
Hypertension, Aging	Human	NMR					√		√			4
Induced hypertension	Rat	AAS	×	√	√			√				5
Mg deficient food	Rat	AAS	×	×	×	×		×		√		6
Mg deficient food	Rat	MAPP	×	×	×	×		×		√	√	7
Migraine	Human	NMR							√			8
Preeclampsia	Human	NMR					√		√			9
Traumatic brain injury	Rat	NMR							√			10

√ and ×show the Mg indexes that have been tested in each research. √ means the Mg index effectively responding to the modification of experimental conditions. ×means no response.

*, please see abbreviations as below:

AAS, atomic absorption spectroscopy; ICP-AES, inductively coupled plasma atomic emission spectrometry; MAPP, Magnesium ammonium phosphate precipitate; NMR, nuclear magnetic resonance.

Supplementary Table S2: Mg detection in blood cells

Research field	Species	Assay tech.*	CSF	Urine	Serum	Plasma	Plasma ionized	RBC ionized	RBC membrane	RBC total	Lymphocyte total	Lymphocyte ionized	Platelet total	24h retention	Reference
Aging, hypertension, diabetes	Human	NMR						√							11
Angina	Human	CC, AAS		√	×					√	√			√	12
Anxiety/stress	Human	AES		√											13
Congestive heart failure	Human	AAS			×						×				2
Diabetes	Human	NMR						√							14
Genetic low Mg, Mg deficient food	Mouse	AAS		√		√				√					3
Hemodialysis	Human	AAS, ISE, FP				√	√			√	√	√			15
Hypertension	Human	AAS				×			√	×					16
Hypertension	Human	FP										×			17
Hypertension	Human	NMR				×		√							18
Hypertension	Human	NMR						√							19
Hypertension	Human	NMR						√							20
Hypertension	Human	FP										×			21
Hypertension, insulin inducement	Human	AAS, NMR						√		√					22
Hypertension, obesity	Human	DCPS				×				√			√		23

Research field	Species	Assay tech.*	CSF	Urine	Serum	Plasma	Plasma ionized	RBC ionized	RBC membrane	RBC total	Lymphocyte total	Lymphocyte ionized	Platelet total	24h retention	Reference
Hypomagnese-mia	Human	AAS			√						×				24
Induced hypertension	Rat	AAS								√					5
Insulin inducement	Human	NMR						√							25
Maternal and cord blood	Human	NMR, ISE					√	√							26
Mg deficient food	Rat	AAS				√									6
Mg deficient food	Rat	MAPP			√										7
Mg deficient food	Rat	CC				√									27
Mg deficient food	Calve	AAS	√			√				√					28
Mg deficient food	Rat	Isotope, ICP-MS				√	√			√	×		×		29
Migraine	Human	AAS, FP				×				√	×	√			30
Oral Mg treat	Rat	CC	√												31
Sleep deprivation	Rat	AAS, ISE				×	√								32

√ and × show the Mg indexes that have been tested in each research. √ means the Mg index effectively responding to the modification of experimental conditions. × means no response.

*, please see abbreviations as below:

AAS, atomic absorption spectroscopy; AES, atomic emission spectrometry; CC, calmagite chromometry; DCPS, direct current plasma spectrometer; FP, fluorescence probe; ICP-MS, inductively coupled plasma mass spectrometry; ISE, ion-selective electrode; MAPP, Magnesium ammonium phosphate precipitate; NMR, nuclear

magnetic resonance.

References

1. Andrasi, E., Pali, N., Molnar, Z. & Kosel, S. Brain aluminum, magnesium and phosphorus contents of control and Alzheimer-diseased patients. *J Alzheimers Dis* **7**, 273-284 (2005).
2. Ralston, M.A. *et al.* Magnesium content of serum, circulating mononuclear cells, skeletal muscle, and myocardium in congestive heart failure. *Circulation* **80**, 573-580 (1989).
3. Feillet-Coudray, C. *et al.* Magnesium metabolism in mice selected for high and low erythrocyte magnesium levels. *Metabolism* **53**, 660-665 (2004).
4. Resnick, L.M. *et al.* Direct magnetic resonance determination of aortic distensibility in essential hypertension: relation to age, abdominal visceral fat, and in situ intracellular free magnesium. *Hypertension* **30**, 654-659 (1997).
5. Senturk, U.K., Kaputlu, I., Gunduz, F., Kuru, O. & Gokalp, O. Tissue and blood levels of zinc, copper, and magnesium in nitric oxide synthase blockade-induced hypertension. *Biol Trace Elem Res* **77**, 97-106 (2000).
6. Martindale, L. & Heaton, F.W. Magnesium deficiency in the adult rat. *Biochem J* **92**, 119-126 (1964).
7. Watchorn, E. & McCance, R.A. Subacute magnesium deficiency in rats. *Biochem J* **31**, 1379-1390 (1937).
8. Lodi, R. *et al.* Deficit of brain and skeletal muscle bioenergetics and low brain magnesium in juvenile migraine: an in vivo 31P magnetic resonance spectroscopy interictal study. *Pediatr Res* **42**, 866-871 (1997).
9. Resnick, L.M. *et al.* Cellular-free magnesium depletion in brain and muscle of normal and preeclamptic pregnancy: a nuclear magnetic resonance spectroscopic study. *Hypertension* **44**, 322-326 (2004).
10. Heath, D.L. & Vink, R. Optimization of magnesium therapy after severe diffuse axonal brain injury in rats. *J Pharmacol Exp Ther* **288**, 1311-1316 (1999).
11. Barbagallo, M., Gupta, R.K., Dominguez, L.J. & Resnick, L.M. Cellular ionic alterations with age: relation to hypertension and diabetes. *J Am Geriatr Soc* **48**, 1111-1116 (2000).
12. Satake, K., Lee, J.D., Shimizu, H., Ueda, T. & Nakamura, T. Relation between severity of magnesium deficiency and frequency of anginal attacks in men with variant angina. *J Am Coll Cardiol* **28**, 897-902 (1996).
13. Grases, G. *et al.* Anxiety and stress among science students. Study of calcium and magnesium alterations. *Magnes Res* **19**, 102-106 (2006).
14. Barbagallo, M., Resnick, L.M., Dominguez, L.J. & Licata, G. Diabetes mellitus, hypertension and ageing: the ionic hypothesis of ageing and cardiovascular-metabolic diseases. *Diabetes Metab* **23**, 281-294 (1997).
15. Huijgen, H.J. *et al.* Intracellular and extracellular blood magnesium fractions in hemodialysis patients; is the ionized fraction a measure of magnesium excess? *Clin Chem* **44**, 639-648 (1998).
16. Kosch, M. *et al.* Alterations in calcium and magnesium content of red cell membranes in patients with primary hypertension. *Am J Hypertens* **14**, 254-258 (2001).

17. Delva, P. *et al.* Intralymphocyte free magnesium and calcium and insulin tolerance test in a group of essential hypertensive patients. *Life Sci* **63**, 1405-1415 (1998).
18. Dominguez, L.J., Barbagallo, M., Sowers, J.R. & Resnick, L.M. Magnesium responsiveness to insulin and insulin-like growth factor I in erythrocytes from normotensive and hypertensive subjects. *J Clin Endocrinol Metab* **83**, 4402-4407 (1998).
19. Resnick, L.M., Gupta, R.K. & Laragh, J.H. Intracellular free magnesium in erythrocytes of essential hypertension: relation to blood pressure and serum divalent cations. *Proc Natl Acad Sci U S A* **81**, 6511-6515 (1984).
20. Resnick, L.M., Gupta, R.K., Gruenspan, H., Alderman, M.H. & Laragh, J.H. Hypertension and peripheral insulin resistance. Possible mediating role of intracellular free magnesium. *Am J Hypertens* **3**, 373-379 (1990).
21. Delva, P.T., Pastori, C., Degan, M., Montesi, G.D. & Lechi, A. Intralymphocyte free magnesium in a group of subjects with essential hypertension. *Hypertension* **28**, 433-439 (1996).
22. Barbagallo, M., Dominguez, L.J., Tagliamonte, M.R., Resnick, L.M. & Paolisso, G. Effects of glutathione on red blood cell intracellular magnesium: relation to glucose metabolism. *Hypertension* **34**, 76-82 (1999).
23. Corica, F. *et al.* Changes in plasma, erythrocyte, and platelet magnesium levels in normotensive and hypertensive obese subjects during oral glucose tolerance test. *Am J Hypertens* **12**, 128-136 (1999).
24. Sacks, G.S. *et al.* Mononuclear blood cell magnesium content and serum magnesium concentration in critically ill hypomagnesemic patients after replacement therapy. *Nutrition* **13**, 303-308 (1997).
25. Barbagallo, M., Gupta, R.K. & Resnick, L.M. Cellular ionic effects of insulin in normal human erythrocytes: a nuclear magnetic resonance study. *Diabetologia* **36**, 146-149 (1993).
26. Bardicef, M. *et al.* Perinatal cellular ion metabolism: 31P-nuclear magnetic resonance spectroscopic analysis of intracellular free magnesium and pH in maternal and cord blood erythrocytes. *J Soc Gynecol Investig* **3**, 66-70 (1996).
27. Bardgett, M.E., Schultheis, P.J., McGill, D.L., Richmond, R.E. & Wagge, J.R. Magnesium deficiency impairs fear conditioning in mice. *Brain Res* **1038**, 100-106 (2005).
28. Reynolds, C.K., Bell, M.C. & Sims, M.H. Changes in plasma, red blood cell and cerebrospinal fluid mineral concentrations in calves during magnesium depletion followed by repletion with rectally infused magnesium chloride. *J Nutr* **114**, 1334-1341 (1984).
29. Feillet-Coudray, C., Coudray, C., Gueux, E., Mazur, A. & Rayssiguier, Y. A new in vitro blood load test using a magnesium stable isotope for assessment of magnesium status. *J Nutr* **133**, 1220-1223 (2003).
30. Thomas, J. *et al.* Free and total magnesium in lymphocytes of migraine patients - effect of magnesium-rich mineral water intake. *Clin Chim Acta* **295**, 63-75 (2000).
31. Slutsky, I. *et al.* Enhancement of learning and memory by elevating brain magnesium. *Neuron* **65**, 165-177 (2010).

32. Akanmu, M.A., Meludu, S.C. & Honda, K. Effects of 6-hour total sleep deprivation on plasma magnesium and calcium levels in rats. *Sleep and Biological Rhythms* **1**, 97-100 (2003).
